# Two new species of *Archaeohelorus* (Hymenoptera, Proctotrupoidea, Heloridae) from the Middle Jurassic of China

**DOI:** 10.3897/zookeys.369.6561

**Published:** 2014-01-13

**Authors:** Xiaoqing Shi, Yunyun Zhao, Chungkun Shih, Dong Ren

**Affiliations:** 1 College of Life Sciences, Capital Normal University, 105 Xisanhuanbeilu, Haidian District, Beijing 100048, China

**Keywords:** Fossil wasps, Heloridae, *Archaeohelorus*, Middle Jurassic, China

## Abstract

Two new fossil species, *Archaeohelorus polyneurus* **sp. n.** and *A. tensus* **sp. n.**, assigned to the genus *Archaeohelorus* Shih, Feng & Ren, 2011 of Heloridae (Hymenoptera), are reported from the late Middle Jurassic, Jiulongshan Formation of Inner Mongolia, China. Based on the well-preserved forewings and hind wings of these specimens, the diagnosis of the *Archaeohelorus* is emended: forewing 2cu-a intersecting Cu and Rs+M at the same point or postfurcal, and hind wing may have tubular veins C, Sc+R, R, Rs, M+Cu, M and Cu distinct, or simplified venation. The new findings also elucidate the evolutionary trend of forewing and hind wing venation and body size for the Heloridae from the late Middle Jurassic to now.

## Introduction

Proctotrupoidea Latreille, 1802, including 11 extant families, is a significant group within Hymenoptera for their long evolutionary history, special morphology and diversity (Grimaldi and Engel 2005). Most extant species of Proctotrupoidea are small wasps except for the giants of the family Pelecinidae (Shih et al. 2010). Heloridae, a small family in Proctotrupoidea, have the earliest fossil records from the late Middle Jurassic (Shih et al. 2011).

Up to date, fossil Heloridae contains 8 genera and 12 species, which have been summarized by Shi et al. (2012). These species have been described from the late Middle Jurassic of Daohugou Ningcheng, China (Shih et al. 2011); the Late Jurassic of Karatau, Russia (Rohdendorf 1938), and of Laiyang, China (Zhang 1992); the Early Cretaceous of Turga, Russia (Rasnitsyn 1990), of Beipiao, China (Zhang and Zhang 2001; Shi et al. 2012); and of Gurvan-Ereny-Nuru, Western Mongolia (Rasnitsyn 1986).

So far, 7 species of Heloridae from China have been reported, including *Archaeohelorus hoi* Shih, Feng & Ren, 2011; *Gurvanhelorus beipiaoensis* Shi, Shih & Ren, 2012; *Protocyrtus validus* Zhang & Zhang, 2001; *Sinohelorus elegans* Shi, Shih & Ren, 2012; *Spherogaster coronata* Zhang & Zhang, 2001; and *Spherogaster saltatrix* Shi, Shih & Ren, 2012.

Extant helorids contains only one genus, *Helorus* Latreille, 1802, with 12 known valid species mostly in the Holarctic Region. They are parasitoids of larvae of chrysopid lacewings (Neuroptera: Chrysopidae: Chrysopinae: *Chrysopa* species) (van Achterberg 2006). Among the known species, only *Helorus chinensis* He, 1992 was described from China (He 1992).

Recently, we collected two well-preserved fossil specimens referable to Heloridae from the late Middle Jurassic Jiulongshan Formation at Daohugou Village, Ningcheng County, Inner Mongolia, China. Based on these new findings, the diagnosis of *Archaeohelorus* Shih, Feng & Ren, 2011 is emended and two new species, *Archaeohelorus polyneurus* sp. n. and *Archaeohelorus tensus* sp. n., are described. This is the second report of Heloridae in the late Middle Jurassic.

The age of the Daohugou fossil-bearing beds is interpreted to be late Middle Jurassic (*ca* 165 Ma; Ren et al. 2010). This deposit is interpreted to have accumulated in streams and lakes within a humid and warm-temperate climate (Ren et al. 2002). It is rich in well-preserved fossils, especially a high level of insect diversity have been reported including Ephemeroptera (Huang et al. 2008), Odonata (Li et al. 2011), Plecoptera (Liu et al. 2011), Blattodea (Wei et al. 2012), Orthoptera (Gu et al. 2012), Homoptera (Wang et al. 2012), Heteroptera (Lu et al. 2011), Neuroptera (Wang et al. 2010; Shi et al. 2011), Raphidioptera (Engel & Ren, 2008), Coleoptera (Tan et al. 2012), Mecoptera (Ren et al. 2009; Wang et al. 2012), Hymenoptera (Wang et al. 2012), and Diptera (Liu et al. 2012).

## Materials and methods

All the materials have been collected near Daohugou Village, Shantou Township, Ningcheng County, Inner Mongolia, China; the late Middle Jurassic (Bathonian-Callovian boundary, 165 Ma). All fossil specimens are housed in the Key Lab of Insect Evolution & Environmental Changes, College of Life Sciences, Capital Normal University, Beijing, China.

The specimens were examined dry or under alcohol using a M165 C dissecting microscope (Leica) and are illustrated with the aid of a drawing tube attachment. The figures were drawn by Adobe Photoshop CS5 and CorelDraw 12.0. Morphological terminology and the system used here follow those of Huber and Sharkey (1993) and Rasnitsyn and Zhang (2010).

## Systematic Paleontology

### Class Insecta L., 1758
Order Hymenoptera L., 1758
Suborder Apocrita Gerstaecker, 1867
Superfamily Proctotrupoidea Latreille, 1802
Family Heloridae Foerster, 1856
Subfamily Mesohelorinae Rasnitsyn, 1990

#### Genus Archaeohelorus

Shih, Feng & Ren, 2011

http://species-id.net/wiki/Archaeohelorus

##### Type species.

*Archaeohelorus hoi* Shih, Feng & Ren, 2011

##### Emended diagnosis.

Forewing 2cu-a intersecting Cu and Rs+M at the same point or postfurcal. Hind wing may have tubular veins C and Sc+R separated at base, R developed, M+Cu robust and forking at the basal part, M and Cu distinct, or simplified venation.

##### Species included.

*Archaeohelorus hoi* Shih, Feng & Ren, 2011, *Archaeohelorus polyneurus* sp. n. and *Archaeohelorus tensus* sp. n.

##### Remarks.

This genus was established by Shih et al. 2011 based on a holotype, allotype and six paratypes from the Middle Jurassic of Daohugou, Inner Mongolia, China. Due to lack of discernible hind wings on the fossils of holotype and allotype, the hind wing venation was not described. With the new forewing and hind wing venational information on our new materials, we emended the generic diagnosis.

#### Archaeohelorus polyneurus sp. n.

http://zoobank.org/BFECD83C-516A-4F08-BBB3-72A5EB6DFF41

http://species-id.net/wiki/Archaeohelorus_polyneurus

Figures 1 , 2

##### Etymology.

The specific nameis from Greek word “*polyneurus*”, means “many veins”, referring to the complete venation of the hind wing preserved.

##### Type material.

Holotype, CNU-HYM-NN2012052, dorsal view, gender unknown. A well-preserved body with almost complete forewings and hind wings and part of legs, but head missing. Paratype: CNU-HY-NN2008010, dorsal view, a well-preserved almost complete body with forewings and right hind wing and part of legs, previously mis-identified as a paratype of *Archaeohelorus hoi*.

##### Locality and age.

Jiulongshan Formation, Middle Jurassic, Daohugou Village, Shantou Township, Ningcheng County, Inner Mongolia, China.

##### Diagnosis.

In forewing, 2cu-a intersecting Cu and Rs+M at the same point and cell r obtuse-angled triangular (vs. approximately right-angled triangular in *Archaeohelorus hoi*). Hind wing with Sc+R confluent with C and extended to costal margin, R developed. M+Cu distinct and forking at the basal part, M and Cu robust.

##### Remarks.

Upon further examination, we found that a paratype of *Archaeohelorus hoi*, CNU-HYM-NN2008010, has forewing 2cu-a intersecting Cu and Rs+M at the same point and cell r obtuse-angled triangular (vs. approximately right-angled triangular in *Archaeohelorus hoi*); and hind wing with tubular vein C parallel with Sc+R at base, M+Cu robust and forking at the basal section, M long, 1-Cu, 2-Cu distinct. These venational characters are consistent with the diagnostic characters of *Archaeohelorus polyneurus* sp. n., hence we transfer CNU-HYM-NN2008010 as a paratype of *Archaeohelorus polyneurus* sp. n.

##### Description of holotype.

**Figure 1.**
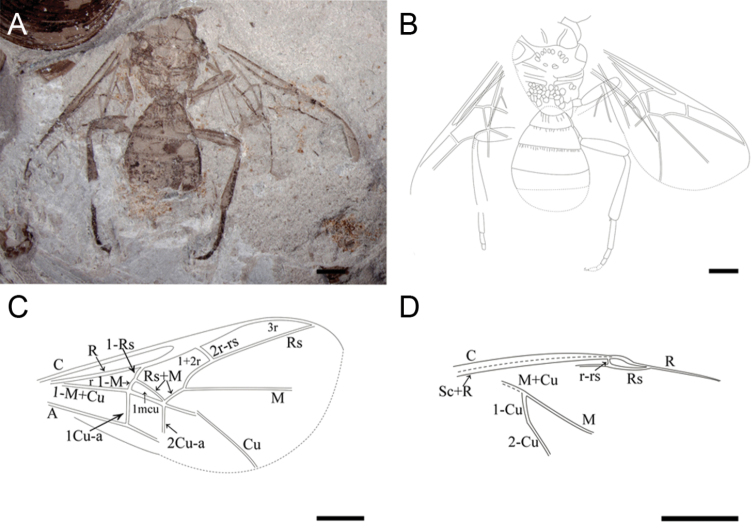
Holotype of *Archaeohelorus polyneurus* sp. n. CNU-HYM-NN-2012052. **A** Photo **B** line drawing **C** line drawing of forewing **D** line drawing of hind wing. Scale bars: 1 mm. (Online figure in color.)

A medium-sized body with both forewings and hind wings well-preserved, but, without head (Fig. 1). Forewing broad. Mesosoma suboval, nearly 1.35 times as long as wide; mesoscutum trapezoidal with notauli distinct and concave; tegula triangular; scutellum broad with two rows of pits; metanotum relatively wide with plenty of pits; propodeum transverse, 2.89 times as broad as long.

Metasoma suboval with six segments; first metasomal segment transverse, second segment with several longitudinal ridges; third and fourth segments trapezoidal with shorter longitudinal ridges anteriorly; other ones smooth; the end of terminal segment not preserved.

Forewing broad and subtriangular. Pterostigma long and acute apically, not widened beyond mid-length. C robust and extending to apex of forewing. 2r-rs arising from basal one third of pterostigma, slightly oblique apicad, slightly longer than pterostigmal width. R robust, cell r obtuse-angled triangular and closed by R, M+Cu, 1-Rs and 1-M; Rs straight and intersecting with distal part of C. Cell 1+2r six-sided and surrounded by R, 1-Rs, 1-Rs+M, 2-Rs+M, 2-Rs, and 2r-rs. M+Cu straight and distinct; M and Cu distinct, M straight, Cu break after 1m-cu. 1-Rs as long as 1-M and slightly inclined toward wing base. Cell 1mcu small and subtriangular. M and Rs branching at 30% from 1m-cu of the length between 1m-cu and 2r-rs. 1cu-a and 2cu-a distinct and reaching A. 1cu-a in line with 1-M; and 2cu-a intersecting Cu and Rs+M at the same point.

Hind wing with tubular veins C parallel with Sc+R at base; R developed and Rs preserved; M+Cu robust and forking at the basal section, M and 1-Cu long and distinct, 2-Cu long.

Right foreleg with only coxa and femur partially preserved. Right midleg with partial trochanter, spindle-shaped femur and partial tibia preserved. Right hindleg with partial coxa, trochanter trapezoidal and small, robust spindle-shaped femur, long tibia swollen distally with spurs, tarsi with five segments, basitarsus longest and two claws fixing the end of pretarsus.

**Measurements (in mm).** Mesosoma length 3.36, width 2.48; metasoma > 3.38 long; lengths of the first to fifth metasomal segments are 0.34, 0.51, 0.72, 0.94, and 0.55; forewing length 6.52, width > 3.05.

##### Description of paratype.

**Figure 2.**
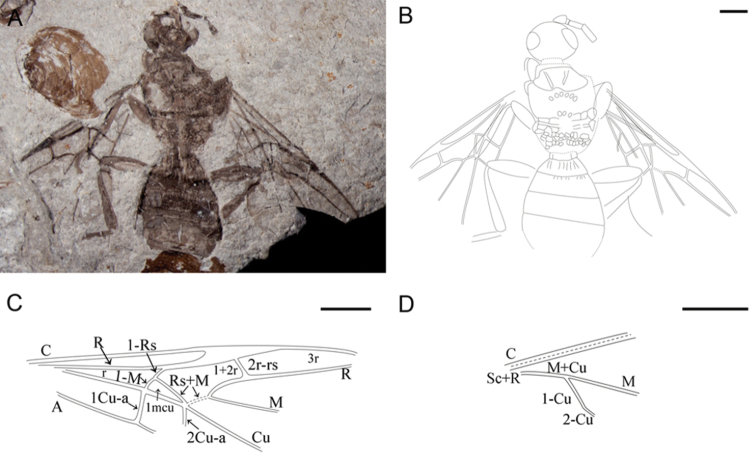
Paratype of *Archaeohelorus polyneurus* sp. n. CNU-HYM-NN-2008010. **A** Photo **B** line drawing **C** line drawing of forewing **D** lLine drawing of hind wing. Scale bars: 1 mm. (Online figure in color.)

Body medium-sized (Fig. 2). Head oval and antennae filiform and thick, scape swollen and bell-shaped, only first and second flagellomeres preserved. Mesosoma, forewing and metasoma same as holotype, the end of terminal segment not preserved. Hind wing with tubular vein C parallel with Sc+R at base, M+Cu robust and forking at the basal section, M long, 1-Cu, 2-Cu distinct. Left foreleg and right midleg with only coxa and femur partially preserved. Left midleg with femur robust and tibia long, left hindleg with femur robust and spindle-shaped, long tibia swollen distally. Right hindleg with femur robust and spindle-shaped, tibia partially preserved.

**Measurements (in mm).** Body length > 14.6; head length 1.89, width 1.53; mesosoma length 3.58, width 2.52; metasoma length 3.68; forewing length > 6.54, width > 2.88.

##### Comparison.

This species can be assigned to *Archaeohelorus* Shih, Feng & Ren, 2011 by its metasoma with six segments and the first segment narrow and transverse. Forewing 1-Rs as long as 1-M. Cell 1mcu small and subtriangular, 1cu-a in line with 1-M; and 2cu-a intersecting Cu and Rs+M at the same point. Compared with *Archaeohelorus hoi*, *Archaeohelorus polyneurus* has preserved hind wing with Sc+R confluent with C and extended to costal margin, R developed. M+Cu distinct and forking at the basal part, M and Cu robust; in forewing, cell r obtuse-angled triangular (vs. approximately right-angled triangular in *Archaeohelorus hoi*). Besides, *Archaeohelorus polyneurus* has a much larger body size than *Archaeohelorus hoi*, length of forewing 6.52 mm or > 6.54 mm (vs. 3.17 mm in *Archaeohelorus hoi*).

#### Archaeohelorus tensus sp. n.

http://zoobank.org/84E4B637-E6E6-4799-929E-1536DA5213A5

http://species-id.net/wiki/Archaeohelorus_tensus

Figure 3

##### Etymology.

The specific name “*tensus*” means stretching and long, referring to the shape of the forewing.

##### Type material.

Holotype, CNU-HYM-NN2012056p/c, part and counterpart, dorsal view, male. A well-preserved almost complete body with antenna, forewings and part of legs and partial hind wings.

**Figure 3.**
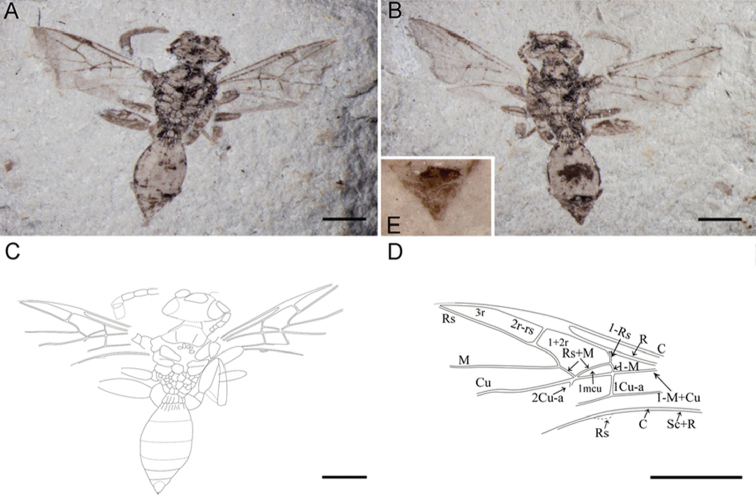
Holotype of *Archaeohelorus tensus* sp. n. CNU-HYM-NN-2012056 p/c, part and counterpart. **A** Photo of part **B** photo of counterpart **C** line drawing of part **D** line drawing of forewing **E** terminal segments of counterpart (under alcohol). Scale bars: 1 mm. (Online figure in color.)

##### Locality and age.

Jiulongshan Formation, Middle Jurassic, Daohugou Village, Shantou Township, Ningcheng County, Inner Mongolia, China.

##### Diagnosis.

Forewing 2cu-a postfurcal with intersection of Cu and Rs+M. First abscissa of Rs (1-Rs) and basal section of M (1-M) arched toward basal of wing. Hind wing with tubular veins C, Sc+R, and Rs preserved.

##### Description.

A smalladult specimen with a total body length of 4.4 mm. Head suboval and large relative to mesosoma in width. Eyes large, and located at the both sides of the head.

Antennae filiform, thick, with 6 segments preserved on left and 5 segments preserved on right. Scape swollen and bell-shaped; pedicel short and quadrate; subsequent flagellomeres not well preserved.

Mesosoma subhexagonal and broader than head; pronotum narrower than head, short, probably covered by mesonotum; mesoscutum distorted in the middle and trapezoidal with notauli not distinct but preserved; tegula large and subtriangular; scutellum round with pits anteriorly; propodeum narrow, transverse and areolated.

Metasoma suboval with seven segments; first metasomal segment transverse with several longitudinal ridges; second segment trapezoidal with less longitudinal ridges anteriorly; other ones smooth; the male terminalia triangular, partially covered by the previous segment.

Forewing broad and subtriangular. Pterostigma long and acute apically, not widened beyond mid-length. C robust and extending near the apex of forewing. 2r-rs arising from basal one third of pterostigma, slightly longer than the width of pterostigma, and slightly oblique apicad. R robust and cell r closed with C, R and 1-RS. Rs straight and intersecting with distal part of C. Cell 1+2r longer and narrower relatively and six-sided surrounded by R, 1-Rs, 1-Rs+M, 2-Rs+M, 2-Rs, and 2r-rs. M+Cu straight and distinct; M and Cu distinct, almost straight. 1-Rs as long as 1-M and slightly inclined toward wing base. Cell 1mcu small, subtriangular and relatively slender with 2-M+Cu 4.3 times as long as 1-M. M and Rs branching at 30% from 1m-cu of the length between 1m-cu and 2r-rs. 1cu-a and 2cu-a distinct and reaching A. 1cu-a in line with 1-M; and 2cu-a postfurcal with Rs+M distinctly.

Hind wing, with tubular veins C parallel with Sc+R at base, Rs short, intersecting distal part of C.

Left foreleg, midleg and hindleg and right foreleg with only coxa and femur partially preserved, hind femur much thicker, nearly three times as long as wide; right midleg with partial spindle-shaped femur and partial tibia preserved, right hindleg with partial coxa, trapezoidal trochanter, robust spindle-shaped femur and relatively thin tibia and some parts of tarsus preserved.

**Measurements (in mm):** Body length 4.67, head length1.39, width 0.68, mesosoma length 1.68, width 1.36; metasoma 2.23 long; lengths of metasomal segments are 0.20, 0.39, 0.48, 0.38, 0.25 and 0.32; forewing length > 2.96, width > 1.36.

##### Remarks and comparison.

This species is assigned to *Archaeohelorus* Shih, Feng & Ren, 2011 by its seven separated metasomal segments, the first metasomal segment transverse with several longitudinal ridges. Forewing 1-Rs as long as 1-M. Cell 1mcu small and subtriangular, 1cu-a in line with 1-M. Compared with *Archaeohelorus hoi*, *Archaeohelorus tensus* has preserved hind wing with tubular veins C, Sc+R, R, and Rs preserved; in forewing, 2cu-a distinctly postfurcal with intersection of Cu and Rs+M (vs. 2cu-a intersecting Cu and Rs+M at the same point in *Archaeohelorus hoi*); cell 1mcu distinctly slender, 4.3 times as long as wide (vs. 2.4 times in *Archaeohelorus hoi*); 1-Rs and 1-M arched toward basal of wing (vs. 1-Rs and 1-M straight in *Archaeohelorus hoi*). It also differs from *Archaeohelorus polyneurus* by its postfurcal 2cu-a, slender cell 1mcu.

## Discussion

In the vast and extensive Daohugou fossil insect collection (>200,000 insect fossil specimens) at the Capital Normal University, only ten helorids are collected so far: two are *Archaeohelorus polyneurus* sp. n., one is *Archaeohelorus tensus* sp. n., and seven specimens are previously described *Archaeohelorus hoi* Shih, Feng & Ren, 2011. It is likely that helorids might have been a very small group in the Middle Jurassic.

As a relict family, helorids have survived from the Middle Jurassic to now. Among all the fossil specimens, only *Archaeohelorus polyneurus* has distinct and complicated hind wing venation, which were unknown before. Therefore, the new findings are important supplement to helorid record and suggest that the trend for the morphological evolution of the Heloridae is as follows:

In the Middle Jurassic, *Archaeohelorus polyneurus* had forewing cell r obtuse-angled triangle, and hind wing venation clear and more complex (C and Sc+R separated at base, M+Cu short and robust, forking at the basal part, M and Cu distinct) and a much larger body (forewing length > 6.52mm). On the other hand, *Archaeohelorus hoi* and *Archaeohelorus tensus* had forewing cell r approximately right-angled triangular, and hind with simplified venation (only C and Sc+R and/or R preserved) and smaller body (forewing length ≈ 3 mm). In the Late Jurassic to the Early Cretaceous, *Spherogaster coronata* Zhang & Zhang, 2001 had hind wing with only C, R and M present, costal area extremely narrow, and a much larger body size with forewing length of 12 mm. In extant helorids, forewing cell r approximately right-angled triangular, and hind wings have tubular veins C and Sc+R and nebulosus veins of M+Cu, M, Cu and A (Goulet and Huber 1993). The body size is small with forewing about 3.3 mm long (van Achterberg 2006).

## Supplementary Material

XML Treatment for Archaeohelorus

XML Treatment for Archaeohelorus polyneurus

XML Treatment for Archaeohelorus tensus
